# Nursing intervention for preventing postpartum depressive symptoms among Chinese women in Japan

**DOI:** 10.1111/jjns.12336

**Published:** 2020-04-06

**Authors:** Qiongai Jin, Emi Mori, Akiko Sakajo

**Affiliations:** ^1^ School of Health Sciences Fujita Health University Toyoake Japan; ^2^ Graduate School of Nursing Chiba University Chiba Japan; ^3^ Faculty of Nursing Musashino University Tokyo Japan

**Keywords:** culture, immigrants, midwifery, nursing, postpartum depression

## Abstract

**Aim:**

We evaluated the effectiveness of a nursing intervention program for Chinese women who are giving birth in Japan to reduce cross‐cultural stressors during the postpartum period and prevent postpartum depressive symptoms.

**Methods:**

A prospective, before‐and‐after study incorporating a longitudinal mixed‐method design was conducted. Thirty‐eight participants underwent this program from August 2016 to July 2017. The program comprised a maternity class, conversation cards, and a social‐network group. Data collection was initially performed using questionnaires administered in the third trimester (T1)—before the intervention—to obtain participants' basic information, stress levels, depressive symptoms, and cognitive appraisals. Then, stress levels, depressive symptoms, and social support were measured during hospitalization after having given birth (T2) and during the first month postpartum (T3). Finally, through semi‐structured interviews, cognitive appraisal, coping, stress, social support, participants' evaluations of the intervention were determined.

**Results:**

Post‐intervention, all participants showed positive cognitive appraisals, although eight also showed some negative appraisals. At T3, 36 participants did not report experiencing stress owing to cross‐cultural stressors. Furthermore, post‐intervention, participants who returned scores that were suggestive of depression remained identical to that at pre‐intervention (21.1%). Among the eight participants who showed postpartum depressive symptoms during T3, seven did not report experiencing cross‐cultural stressors, but did report encountering maternity stressors.

**Conclusion:**

The nursing intervention program may be effective for preventing postpartum depressive symptoms in Chinese women who give birth in Japan. Since this was a pre‐post study in which one group was measured pre‐intervention and again post‐intervention, we did not register in a publicly assessible database.

Postpartum depression is a significant issue worldwide; it has a harmful influence on mothers, children, and families (Glasheen, Richardson, & Fabio, 2009). Although numerous studies have examined intervention methods for preventing postpartum depression and for improving postpartum mental health in mothers from many different countries (Elliott et al., 2000; Gao, Xie, Yang, & Chan, 2015; Sato & Sato, 2010), effective preventive approaches for immigrant women remain scarce. This study measured the effectiveness of a nursing intervention program (NIP) for Chinese women who are giving birth in Japan. The program was designed to reduce the stress such mothers can experience from cross‐cultural stressors during the postpartum period and prevent postpartum depressive symptoms.

## 1 BACKGROUND

Postpartum depressive symptoms are mental health issues that occur in new mothers, generally 1–2 months after delivery, and the incidence rate can range from 0 to 40% depending on nationality, screening time, and the measurement tools used (Halbreich & Karkun, 2006). The prevalence of postpartum depressive symptoms among mainland Chinese women is approximately 19% (Xie, He, Koszycki, Walker, & Wen, 2009); however, it is approximately 54.5% among Chinese women living overseas (Jin, Mori, & Sakajo, 2016).

Risk factors for postpartum depression include a history of mental disorder, discordant relationships, a lack of social support, and stressful life events (Beck, 2001; O'Hara & Swain, 1996; Yim, Stapleton, Guardino, Hahn‐Holbrook, & Schetter, 2015). However, immigrant women can experience additional risk factors, such as a short residence period in a new country, low local language ability, and differences in cultural conventions (Falah‐Hassani, Shiri, Vigod, & Dennis, 2015; Taniguchi & Baruffi, 2007); in fact, there are moderate positive correlations between postpartum depressive symptoms and cross‐cultural stressors (*r* = .527; Jin et al., 2016). For instance, Chinese women practice a special post‐childbirth tradition called “*zuoyuezi*,” which is designed to help recovery after childbirth, increase milk supply, and recondition their bodies (Cheung, 1997). This traditional practice is an essential rite of passage in China, and it features rules for the first postpartum month regarding maternal meals, clothes, mental well‐being, and other areas. For example, new mothers should receive assistance from their family members or a paid nurse, who should ensure mothers' smooth recuperation, help them adapt to motherhood, and perform housework in their stead. Moreover, postpartum mothers should avoid all cold substances (e.g., cold water, winds, foods), and should even brush their teeth and shower with warm or pre‐boiled water only. However, performing *zuoyuezi* is difficult in Japan because it requires substantial support during the first postpartum month. Thus, considering cultural values is important when formulating a preventive program for postpartum depressive symptoms.

The need to address postpartum depressive symptoms is clear; however, effective preventive approaches for immigrant women are scarce. Scant interventions for preventing perinatal depressive symptoms among immigrant women giving birth in foreign countries currently exist (Jesse et al., 2015; Le, Perry, & Stuart, 2011); however, despite their effectiveness for decreasing depressive symptoms during pregnancy, their effectiveness regarding preventing postpartum depressive symptoms remains unclear.

Considering this, we devised a unique NIP based on Lazarus and Folkman's (1984) transactional theory of stress. Based on this theory, we assumed that Chinese women tend to adopt negative perspectives of cross‐cultural and maternity stressors, such as becoming mothers and raising children in Japan, and consequently cope poorly with their situations. Owing to this failure to cope, stress responses can be developed, which can then lead to depressive symptoms. Therefore, to prevent postpartum depressive symptoms, a suitable NIP should focus on cognitive appraisal and coping behaviors. We felt that a combination of methods would improve the effect of the intervention, as postpartum depressive symptoms develop through the influence of multiple risk factors (Arai & Takahashi, 2006).

## 2 METHODS

### 2.1 Aims

We evaluated the effectiveness of a NIP designed to reduce the stress caused by cross‐cultural stressors during the postpartum period, and thereby prevent postpartum depressive symptoms in Chinese women who are giving birth in Japan. We hypothesized that the following would happen to Chinese women who attended this NIP: (a) their cognitive appraisals at the first month postpartum would be more positive than before the NIP; (b) they would not report experiencing stress owing to cross‐cultural stressors; and (c) the percentage with depressive symptoms would not increase from pre‐ to post‐NIP.

### 2.2 Design

This study was conducted between August 2016 and July 2017 using a single‐experiment, pretest‐posttest design. The approach was primarily quantitative (self‐report questionnaires) with a qualitative component (semi‐structured interview). Data were collected at three different points: during the third trimester, but before NIP (T1); during the postpartum hospital stay, after NIP (T2); and during the first month postpartum, after NIP (T3). To collect data on time, the first author sent reminders via a short email to participants to confirm or arrange interview time slots. This study is an offshoot of the first author's dissertation, and the complete protocol of the larger study is available elsewhere (Jin, 2018). Regarding sample size, seeking 80% power at a 5% significance level, we determined that at least 38 participants were required; however, taking potential dropouts into consideration, we eventually recruited 63 mothers.

### 2.3 Participants

Participants were recruited from the obstetric outpatient clinic of four general hospitals in the greater Tokyo area. Nurses selected potential participants at each hospital using medical records. Then, the first author, who could communicate with participants in Chinese, invited the women to participate after they were explained the study details and provided informed written consent. The inclusion criteria were: (a) being a pregnant Chinese woman who planned to give birth in Japan; (b) being at least 30 weeks pregnant with a singleton pregnancy and predicted to have a normal pregnancy and delivery; (c) intending to give birth at the facility providing maternal care; (d) having the ability to read and understand Chinese; (e) being married or having a partner; and (f) being willing to participate in the entire NIP. The exclusion criteria were: (a) having a personal or family history of mental illness; and (b) having previously given birth prematurely or to a low‐birth‐weight infant.

As China's one‐child policy was in place for several decades (albeit relaxed in 2016), it is likely that many Chinese women previously did not consider raising a second child. Thus, we believed that raising two or more children may be a source of stress for multiparas giving birth in Japan. Furthermore, since the length of stay in a foreign country is related to postpartum depression (Falah‐Hassani et al., 2015), we deemed that including all such women as participants was necessary to determine the s comprehensive ability of the NIP to prevent postpartum depressive symptoms.

### 2.4 Intervention

The NIP comprised a maternity class, conversation cards, and a social‐network group. It was offered in addition to standard nursing care. A summary of the program is provided in Table 1; the complete protocol of the NIP is described in a previous study (Jin, 2018). The design of the NIP was based on a previous systematic review (Arai & Takahashi, 2006), which suggested that a combination of providing information, fostering psychological/social adaptation to maternal roles, and teaching coping methods is effective for preventing postpartum depressive symptoms.

**Table 1:** Nursing intervention program (in Chinese)

Module title	Module description
Maternity class (3rd trimester)	Approximately 90 min of group education, with a pamphlet (Picture 1): Changes in physical and mental condition after childbirth Difference between traditional Chinese practice and modern Japanese practice, plus coping methods Childcare arrangements
Conversation cards (for use during the postpartum hospital stay)	For seeking support from nurses for common issues, simply by showing them the relevant card. The lesson in the maternity class explained how the participants should use the cards, and a role play was performed (Picture 2).
Social‐network group (3rd trimester~1st month postpartum)	A tool that allowed the Chinese mothers to obtain peer support by communicating through the internet regarding postpartum life, childcare, troubles, etc. A social‐network group was created for each class group and participants were given instruction on how to use the tool.

For the maternity class, each participant received one 90‐minute class during their third trimester; there were 19 classes altogether, each featuring a group of 2–4 women. The classes were held at a time that was convenient for the women involved, and during their prenatal checkup at the facility where they were enrolled in prenatal care and intending to give birth. In the class, we distributed a Chinese‐language pamphlet which provided information on postpartum life, including changes in physical and mental conditions after childbirth, differences between traditional Chinese practice and modern Japanese practice, coping methods, and childcare arrangements. The class was instructed by a Chinese researcher—the first author, a PhD candidate who had trained in giving maternity classes in advance. A Japanese midwife was present to respond to specific questions, such as the best means of spending the puerperium period in Japan, and to provide specialized responses to obstetrics‐related problems.

The conversation cards were given to the participants during the maternity class to help them more easily obtain assistance from Japanese nurses during their hospitalization after delivery. The class also featured explanations regarding means of using the conversation cards, and the participants then performed role play with the Japanese midwife. The content of the cards was prepared based on stressful episodes previously highlighted (Jin et al., 2016); and the questions were based on those that are frequently asked clinically, which were identified with the cooperation of two midwives who had 5 or more years of clinical experience. Additionally, answer cards for nurses were prepared, which participants could give to nurses so that they could respond to participants' questions in Chinese.

The social‐network groups were created using WeChat® (www.wechat.com), a chat tool that facilitates communication through an internet browser, with each featuring the participants who attended the same maternity class. There is a high likelihood that sharing experiences, such as childcare anxiety and postpartum conflict within a peer group is beneficial for mothers' well‐being, and has positive effects on achieving motherhood, increasing confidence, and reducing fear and anxiety in mothers (Manaka, 2016). Accordingly, we assumed that cognitive evaluations of cross‐cultural and maternity stressors would be positively changed owing to sharing daily life experiences and exchanging childcare information with peers during the puerperal period. In addition, for some groups, the peers included multiparas who had given birth in Japan before, which ensured a diversity of resources and allowed the new mothers to receive information regarding selecting the most appropriate method of addressing stressors. Participants who attended the entire nursing program and agreed to undergo a final interview received a nursery item as a gift (¥500).

### 2.5 Data collection

**Figure 1:**
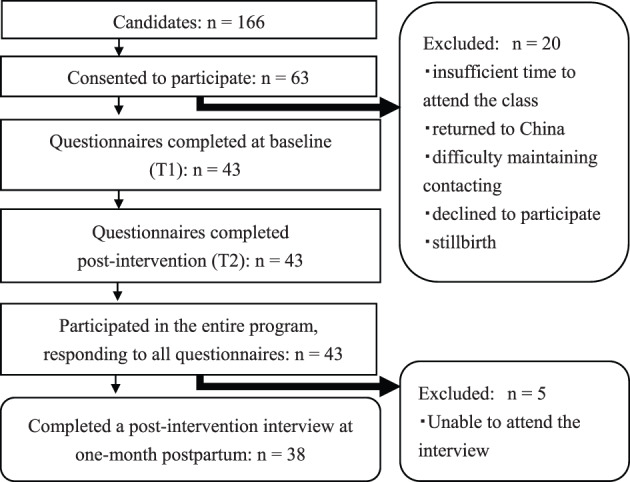
Flow diagram showing the number of participants at each stage of the process

Data were collected between August 2016 and July 2017. Overall, based on the inclusion and exclusion criteria, 166 potential participants were approached by the nurses at each hospital. (Figure 1). Then, they were given an envelope that included an outline of the program, a questionnaire, and an informed consent form that they were asked to complete and return via mail.

The baseline data collection questionnaire (T1) included sociodemographic questions, the State–Trait Anxiety Inventory (STAI; Zheng et al., 1993), and the Edinburgh Postnatal Depression Scale (EPDS; Lee et al., 1998). Post‐NIP quantitative data (T2) were collected during the postpartum hospital stay, including maternal and fetal data during labor, and Social Support Scale (SSS; Chen et al., 2011), and STAI and EPDS responses. The final follow‐up questionnaire (T3) was administered 1 month after the NIP ended, which included maternal and fetal data at 1 month postnatal, as well as SSS, STAI, and EPDS scores. In addition, an interview (T3) of approximately 30 min was conducted, which focused on social status, cognitive appraisal, coping behaviors, and stressful episodes.

A Chinese version of the demographic survey was developed by the first author. The questionnaire data concerned the following, regarding both participants and their partners: age, education level, nationality, years of residency, immigration status, work, family composition, annual family income, and puerperal history. Moreover, along with the progress of pregnancy and childbirth, data regarding children's health status were also obtained.

### 2.6 Measures

#### 2.6.1 *STAI*

Stress was measured using the Chinese version of the STAI (Zheng et al., 1993), which was originally developed by Spielberger, Gorsuch, and Lushene (1970) and is widely used to measure anxiety in perinatal women. We used the “state anxiety” subscale of the STAI (A‐State) to assess temporary conditions of anxiety before and after childbirth. This subscale comprises 20 statements (e.g., “I feel calm”) and respondents indicate their level of agreement with each based on how they feel at the given moment using a four‐point scale (from 1 = “not at all” to 4 = “very much so”). Total scores thus ranged 20–80. Regarding the internal consistency of the A‐State, the Cronbach's alpha value for this study was .86, which indicates high reliability (Zheng et al., 1993). In a previous study featuring an Australian sample of women in the perinatal period, a cut‐off value greater than 40 on the A‐State scale was determined to yield optimal sensitivity (80.95%) and specificity (79.75%) regarding determining cases of anxiety (Grant, McMahon, & Austin, 2008). Since no previous study has investigated the optimal levels of sensitivity and specificity of the STAI for Chinese women in the perinatal period, we applied the cut‐off values used in the Australian study; that is, a score of >40 indicated high anxiety.

#### 2.6.2 *EPDS*

The Chinese version of the EPDS (Lee et al., 1998) was used to measure participants' depressive symptoms before and after the NIP. This scale has been used to screen the depressive symptoms for pregnant women; however, it was originally designed to screen for postpartum depressive symptoms (Chen et al., 2011; Cox, Holden, & Sagovsky, 1987; Lee et al., 1998). The original version of the 10‐item EPDS (Cox et al., 1987) is scored using a four‐point scale (0 = “no, never” to 3 = “yes, most of the time”). Total scores thus ranged 0–30. Lee et al. (1998) recommended that the sensitivity and specificity of this Chinese version were 82% and 86% with a cut‐off score of 10, respectively. An EPDS score of ≥10 indicates potential depressive illness pre‐ and postpartum.

#### 2.6.3 *SSS*

The Chinese version of the SSS (Chen et al., 2011) was used to measure social support status. The SSS comprises 12 items and is scored using a five‐point scale (1–5). Scores range from 12 to 60; higher scores indicate superior support. The internal consistency values were tested, and the Cronbach's αs were .842 (emotional), .805 (household), and .725 (informational) among Chinese and Vietnamese women who had immigrated to Taiwan (Chen et al., 2011). Consequently, in the current study, the reliability scores for each subscale were .882, .908, and .905, respectively, for Chinese women living in Japan.

#### 2.6.4 *Interviews*

Semi‐structured interviews were conducted in Chinese at 1 month postpartum by the first author. Each interview was personalized and was designed to evaluate the effect of the program qualitatively, based on participants' cognitive appraisals of the stressful events they experienced during the postpartum hospital stay and after discharge and their coping behaviors. To determine changes in cognitive appraisal, at T1, each participant was asked two open‐ended questions: “How do you feel about being a mother and rearing a child in Japan?” and “How do you feel about spending postpartum life in Japan?” Responses were compared with those provided during the postpartum interviews. With participants' consent, interviews were audio‐recorded and then transcribed verbatim by the first author. Lastly, participants who agreed to perform the survey underwent the final interview at their hospital when visiting their doctor for a 1‐month postpartum checkup.

### 2.7 Data analysis

For the individual‐level analysis, the qualitative data were analyzed using the content analysis from Graneheim and Lundman (2004). After each interview, the recordings were transcribed verbatim and read to gain a comprehensive understanding of their contents. Thereafter, meaning units and primary codes were identified, and similar primary codes were classified in more condensed units. The content analysis was initially conducted to explore detailed responses regarding social background, social support status, cognitive appraisal, coping behaviors, and stress. Then, the relevance of all variables regarding the conceptual framework was analyzed using both the qualitative and quantitative data. Finally, in the overall analysis, the results of the individual analysis for each case were integrated, and how the NIP affected cognitive appraisal, coping behavior, stress, and depressive symptoms was evaluated. Analysis of variance and Chi‐square analysis were adopted to assess the effects of NIP. In order to compare the STAI at three points, Shapiro–Wilk's test was used to verify a normal distribution, and the within‐group comparison of temporal changes was verified by one‐way analysis of variance with repeated measures. The EPDS was divided into 10 cut‐off values, and a Chi‐square test was performed to analyze the difference between the subjects with depressive and non‐depressive symptoms. The statistical analyses were performed using SPSS version 22 (IBM Corp., Armonk, NY, USA), and a probability of < .05 was considered significant.

### 2.8 Ethical considerations

The research was approved by the authors' university ethics review board and was approved by each of the participating hospitals from which the participants were recruited. Participants were assured that their participation was voluntary, and that they could withdraw from the study at any point without penalty. All data collected were kept anonymous and confidential.

### 2.9 Validity and reliability

The content analysis was conducted by the first author, and the results of content validity and reliability were verified by all three researchers. The pamphlet for the class was created under the supervision of reproductive health nursing scientists who were also midwives; these supervisors included two co‐authors. When translating the Chinese narrative data into Japanese, the first author and a Chinese professor in nursing science, both of whom were proficient in Japanese, performed back translation (Paegelow, 2008).

## 3 RESULTS

Sixty‐three (38.0%) participants signed the consent form; of these, 43 (25.9%) participated in the entire program and responded to all the questionnaires. However, ultimately, 38 (22.9%) participants completed all parts of the study. The women who declined to participate gave reasons such as insufficient time to attend the maternity class (the most common reason), difficulty maintaining contact, and returned to China.

### 3.1 Baseline characteristics

Participants' baseline sociodemographic characteristics are listed in Table 2. The average age was 31.2 years (SD ± 4.8, range: 20–42) and the average duration of residency in Japan was 69.9 months (SD ± 51, range: 7–182). Most participants (73.7%) were primipara, and three of the 10 multiparas were giving birth for the first time in Japan. Twenty‐five participants had a family member visit from China to provide support after childbirth, and 28 participants had more than two supporters, including their spouse. The average SSS score was 45.7 (SD = 8.0, range 28–60) at T2 and 44.7 (SD = 8.6, range 23–60) at T3.

**Table 2:** Demographic data (*n* = 38)

	Mean ± SD (range), n (%)
Mother's age	31.2 ± 4.8 (20.0–42.0)
Education	
College	11 (29.0)
University	20 (52.6)
Graduate school	7 (18.4)
Residency status	
Work permit^a^	13 (34.2)
Non‐work permit^b^	16 (42.1)
Family permit^c^	9 (23.7)
Residency (months)	69.9 ± 51.0 (7.0–182.0)
Occupation	
Employed	14 (36.8)
Unemployed	24 (63.2)
Japanese language level	
Understand Japanese used in everyday situations	18 (47.4)
Understand basic Japanese	11 (28.9)
Do not understand Japanese at all	9 (23.7)
Spouse: age	35.1 ± 9.4 (20.0–72.0)
Spouse: nationality	
China	32 (84.2)
Japan	6 (15.8)
Spouse: residency (months)	85.2 ± 6.1 (15.0–240.0)
Spouse: employment	
Yes	37 (97.4)
No	1 (2.6)
Annual income (pre‐tax)	
Low (<$40 000)	12
High (>$40 000)	26
Family structure	
Nuclear family	31 (78.9)
Extended family	7 (21.1)
Family visits Japan	
Yes	25
No	13
Postpartum helper	
Spouse only	10
Two or more, including spouse	28

a Work permit (official, researcher, engineer, skilled laborer, etc.).

b Non‐work permit (temporary visitor, trainee, student, dependent, etc.).

c Family permit (permanent resident, spouse or child of Japanese nationals, spouse or child of permanent resident, etc.).

Regarding adherence to *zuoyuezi*, 33 participants reported they intended to observe *zuoyuezi* at baseline (T1) (Table 3); however, only 12 participants performed a portion of *zuoyuezi* during their hospital stay after childbirth (T2). Most women who changed their minds reported that “I changed my mind partially after I participated in the maternity class”, and the others mentioned family disapproval and confidence in Yin‐Yang theory. Furthermore, 23 participants did not practice *zuoyuezi* until 1 month postpartum, and two to three participants said that they were unable to practice *zuoyuezi* owing to a lack of support.

**Table 3:** Practice status of *zuoyuezi* a, and the reasons for doing so/not doing so

			N = 38
	T1^b^	T2^c^	T3^d^
Plan to practice *zuoyuezi*	33		
Do not plan to practice *zuoyuezi*	5		
Practiced some elements of *zuoyuezi*			
I changed my mind partially after I attended the maternity class.		10	11
After the class, I decided not to practice *zuoyuezi*, but my family forbade this. Consequently, I performed some elements of *zuoyuezi*.		1	1
I believe in the Yin‐Yang theory and performed part of *zuoyuezi*.		1	1
Did not practice *zuoyuezi*			
After participating in the maternity class, I decided not to perform *zuoyuezi*.		18	18
I never planned to practice *zuoyuezi*.		5	5
Unable to practice *zuoyuezi* postpartum			
There was no one to provide me with the support needed to practice *zuoyuezi*.		2	1
I was looking after my baby, so I could not perform *zuoyuezi*.		1	1

a A traditional practice for the first postpartum month in China.

b During the third trimester, before the nursing intervention program.

c During the postpartum hospital stay, after the nursing intervention program.

d During the first month postpartum, after the nursing intervention program.

### 3.2 Intervention outcomes

As shown in Table 4, all 38 participants showed positive cognitive appraisals by T3, which supports hypothesis 1. Furthermore, they were aware of the differences between *zuoyuezi* and modern Japanese practice and adopted appropriate coping behaviors to address related confusions and difficulties. Overall, 17 participants held discussions through WeChat®, mainly asking questions regarding child‐rearing and milk, and exchanging information related to procedures for their babies' residence cards. However, 21 participants did not use WeChat®, for reasons such as the low number of people in their group, a lack of free time to spend using a mobile phone, and feeling that they did not need to ask others for help because they consulted with their families about most things. Regarding the conversation cards, only two participants used these during their postpartum hospitalization, both of whom were unable to conduct daily conversations in Japanese. However, seven participants who did not understand Japanese at all did not use the cards, mainly because their family helped them to communicate with medical staff, there was a Chinese nurse at the hospital, there was no card that described what they wanted to discuss, or because their spouse was taking care of them.

**Table 4:** Changes in cognitive appraisals and coping behaviors over time

Change in cognitive appraisals		Implementation of appropriate coping behavior	Participant numbers
T1^a^	T3^b^	T3	
Positive appraisal	Positive appraisal	Coping behaviors based on all three intervention elements: maternity class, conversation cards, and SNS group.	None
Positive/negative appraisal^c^	Positive appraisal		None
Negative appraisal	Positive appraisal		3, 8
Positive appraisal	Positive appraisal	Coping behaviors based on one intervention element: maternity class, conversation cards, or SNS group.	9, 22, 24, 27, 31, 32
Positive/negative appraisal^c^	Positive appraisal		4, 12, 13, 21, 23, 26, 29, 34, 36, 38
Negative appraisal	Positive appraisal		1, 6, 7, 14, 15, 16, 18, 20, 25, 28, 33,37
Positive appraisal	Positive/negative appraisal	Coping behaviors based on one intervention element: maternity class, conversation cards, or SNS group.	5, 17
Positive/negative appraisal	Positive/negative appraisal		2, 10, 11, 35
Negative appraisal	Positive/negative appraisal		19, 30

Abbreviation: SNS, social‐network groups.

a During the third trimester, before the nursing intervention program.

b During the first month postpartum, after the nursing intervention program.

c Indicates cognitive appraisals for which positive and negative coexist.

Regarding stress, no significant difference was observed in average STAI scores pre‐ and post‐NIP (Table 5). However, 36 of the 38 participants did not report experiencing stress owing to cross‐cultural stressors, which partially supports hypothesis 2. Only two participants reported feeling such stress; one mentioned relationship conflict with her Japanese mother‐in‐law, while the other mentioned experiencing a language barrier owing to a lack of communication with Japanese speakers (Table 6). Furthermore, 11 participants reported stress caused by maternity stressors. The following factors were mentioned: a lack of social support for childbirth and child‐rearing from families and/or others; relationship conflict with their Chinese spouse, mother‐in‐law, and so on; and neonatal health problems such as hemolytic jaundice.

**Table 5:** Change in State–Trait Anxiety Inventory (STAI) and Edinburgh Postnatal Depression Scale (EPDS) scores over time (N = 38)

Measure	T1^a^	T2^b^	T3^c^		
	Mean (SD)/n	Mean (SD)/n	Mean (SD)/n	F	*P*
STAI	36.0 (8.2)	37.8 (8.5)	36.5 (8.7)	0.51	.60
EPDS < 10	30	27	30	0.72	
EPDS ≥ 10	8	11	8		

*Note*: Analysis was conducted using analysis of variance (F) and Pearson Chi‐square (*P*) tests.

a During the third trimester, before the nursing intervention program.

b During the postpartum hospital stay, after the nursing intervention program.

c During the first month postpartum, after the nursing intervention program.

**Table 6:** Types of stressors

Cross‐cultural stressors^a^	It is difficult to develop a close relationship with my Japanese mother‐in‐law, even if I try to be kind to her, because she maintains emotional distance from me.
	I still cannot become accustomed to living in Japan, and I am worried because I still do not understand Japanese.
Maternity stressors^b^	My mother intended to come to Japan to take care of me, but she was unable to for various reasons. Eventually my mother‐in‐law came to Japan. We are living together now; this arrangement is causing me stress because we are on bad terms with each other.
	My husband had a recent disagreement with my mother about housework, and it has affected my relationship with my husband. Now I have left home, taking my mother and baby with me.
	I feel stress. Difficulties arise in my family as a result of various things.
	I have been worried about taking care of my baby alone since I was discharged from the hospital.
	My hospital stay was comfortable. Sadly, since then I have experienced stress because my mother and mother‐in‐law have contrasting opinions regarding child rearing.
	My mother‐in‐law did not say anything about whether I should practice *zuoyuezi*, but her interference in childcare since I left hospital has caused me stress.^c^
	I was on bad terms with my mother‐in‐law.
	I was worried that I would not have enough milk, since I am a 35 year's old, which qualifies as late maternity.
	My doctor told me there is no problem with baby's weight and my milk when I visited the hospital for my 1 month checkup after childbirth. However, I still worried about my baby's weight, because it is much lighter than I thought it would be.
	My poor baby's weight is so low compared to one of my friends' babies, who born at approximately the same time in China. This made me feel worried.
	I was discharged alone after childbirth because my baby needed to stay in the hospital for a few days due to jaundice. I was worried about my baby.
	I am worried about taking care of my baby alone, as my mother has returned to China.

a An event that caused stress, as well as occurring when exposed to a different culture during a hospital stay for childbirth and after discharge among Chinese women giving birth in a Japanese culture environment.

b An event that caused stress by becoming a mother, as well as occurring during hospitalization for childbirth and after discharge among Chinese women giving birth in a Japanese culture environment or in China.

c A traditional practice for the first postpartum month in China.

Following the NIP, the percentage of participants who scored over 10 on the EPDS (21.1%) had not increased (Table 5); however, the average score was slightly lower post‐NIP (T1, average score = 7.2, SD = 4.2; T2, average score = 7.1, SD = 4.9; T3, average score = 6.8, SD = 3.8); this supports hypothesis 3. Seven of the eight participants who showed postpartum depressive symptoms at 1 month postpartum did not report stress owing to cross‐cultural stressors but did report stress caused by maternity stressors.

## 4 DISCUSSION

The average age of participants was 3 years older than the average childbearing age in mainland China (28.2 years; Jin et al., 2016). This may be because all Chinese women, including multiparous women, who were preparing to give birth in Japan were eligible for inclusion in this study, irrespective of their parity. Participants' average duration of residency in Japan was similar to that observed in a previous study featuring 22 Chinese women giving birth in Japan (71.0 months; Jin et al., 2016); moreover, the Japanese language skills and household incomes of the participants were also similar to those reported in the previous study (Jin et al., 2016). Consequently, we determined that our participants represented normal Chinese residents in Japan.

### 4.1 Cognitive appraisals

Thirty participants returned positive evaluations for all cognitive appraisals after the NIP, with the remaining eight demonstrating both positive and negative evaluations, which largely confirmed our first hypothesis. The positive evaluations may be owing to taking the maternity class during the puerperal period, in conjunction with other nursing interventions. We provided information to participants through the maternity class to increase their awareness of difficulties they may encounter in their postpartum life, or problems that may arise through contact with a different culture. This prompted them to independently explore methods of solving such difficulties and problems, leading to improvements in their problem‐solving capabilities. However, the result that the post‐interventional cognitive appraisals also included negative evaluations indicates that the NIP did not entirely support participants' self‐adjustment to their expected postpartum lives. In particular, primipara Chinese mothers giving birth in Japan, owing to cultural differences and inexperience of postpartum life, can have more to learn during pregnancy than Japanese mothers. Taken together, this suggests that the information provided through the puerperal maternity class was limited, and it failed to motivate the participants to realistically imagine their postpartum life in hospital and life after discharge, independently collect information in preparation for postpartum life, and prepare for receiving family support. Thus, we believe that future puerperal maternity classes should be divided into two sessions: (a) information provision and examining how pregnant women imagine their own postpartum lives and the kind of postpartum support they want to receive from their families; and (b) checking whether the women are capable of independently preparing for and adjusting to postpartum life, as well as providing information for readjustment.

Of the participants who only showed positive evaluations in the post‐interventional cognitive appraisal, two decided, after taking the puerperal maternity class, that they would not adhere to *zuoyuezi*. However, after strong insistence from their families to practice *zuoyuezi*, they reached positive agreements with their families and performed some degree of *zuoyuezi*. *Zuoyuezi* provides an opportunity for puerperants to adapt to their maternal role, and it may be a predictor of good postpartum health (Lee et al., 2004). However, some *zuoyuezi* practices, including not washing one's body and spending long periods in bed, may be detrimental to the recovery and health of puerperants, and may be obstacles to the development of the parent–child relationship (Falah‐Hassani et al., 2015; Gao, Chan, You, & Li, 2010). Thus, since keeping the mother's body clean and engaging in early postoperative ambulation are essential, it is necessary to promote changes in families' understanding of *zuoyuezi*. This suggests the need to provide classes not only for mothers, but also for families who will be supporting postpartum mothers.

### 4.2 Cross‐cultural stressors

Regarding participants' stress, the average score of the STAI before the NIP was 36 (SD = 8.2). Compared to the average score for the same scale for Japanese pregnant women (41.4, SD = 8.3; Okumura et al., 2015) and for pregnant women in mainland China (43.58, SD = 10.79; Zhou & Li, 2011), the initial anxiety score of our participants was low. Moreover, the average score 1 month postpartum was 37.8, which is slightly lower than the average score of Japanese women 1 month postpartum (39.6, SD = 9.6; Sato, 2006). Thirteen participants exhibited an increase in scores at 1 month postpartum compared to scores before delivery. Eleven of these 13 reported experiencing stress from maternity stressors, but not cross‐cultural stressors. This largely confirmed our second hypothesis. This included struggles in the relationship between their Chinese spouse and mother‐in‐law, child‐rearing anxiety after delivery, and a lack of support from family regarding child‐rearing. These findings are consistent with common postpartum stressors observed in Japanese mothers (Okumura et al., 2015). In this NIP, the puerperal maternity class focused on reducing the stress caused by cross‐cultural stressors, which was pursued by providing simple information regarding neonates and child‐rearing as well as postpartum changes in mind and body. We also provided lectures emphasizing the differences between China and Japan regarding puerperants' activities, as well as self‐care practices. However, for the aforementioned 11 participants, the general knowledge they acquired through our puerperal maternity class was insufficient to help them address their maternity stressors. Thus, when studying Chinese women giving birth in Japan, specific instructions must be given not only regarding cross‐cultural circumstances, but also regarding the maternity issues that mothers commonly face. In particular, regarding the maternity stressors that could occur during postpartum hospitalization and discharge, we consider it necessary to align with the facility where the mother gives birth and facilitate a customized approach in that facility.

### 4.3 Postpartum depressive symptoms

Regarding changes in depressive symptoms, the percentage of individuals who presented with depressive symptoms before the NIP was 21.1%; and, at 1 month postpartum, the percentage remained at 21.1%. This largely confirmed our third hypothesis. This percentage is lower than that for foreign women giving birth in Canada (35.1%; Stewart, Gagnon, Sancier, Wahoush, & Dougherty, 2008), for Japanese women giving birth in Hawaii (31.1%; Taniguchi & Baruffi, 2007), and for Chinese primiparas giving birth in Japan (54.5%; Jin et al., 2016).

### 4.4 Limitations

Although the NIP in this study showed promising results regarding the prevention of postpartum depressive symptoms, there are still several limitations. The preventive effect owing to the intervention should be considered with caution because we did not utilize a control group. In this study, we asked 166 candidates from four different medical facilities to participate; however, only 63 provided consent. Of these, 25 declined to participate, because of a lack of time to participate in the puerperal maternity class, inability to maintain contact after approval, or inability to attend an interview 1 month postpartum, which ultimately reduced the participants to 38. Hence, our results may be biased through convenience sampling. Our participants were more willing to participate in the puerperal maternity class and had more time to spare during pregnancy than at 1 month postpartum. Future research should divide the puerperal maternity class into two sessions and shorten the time required of participants or allow participants to participate whenever they visit the hospital for prenatal care; in addition, more convenient ways of collecting postpartum data could also be included, such as home visits.

## 5 CONCLUSION

We developed an NIP for the prevention of postpartum depression. This was administered to Chinese women in the third trimester who planned to give birth in Japan. Our study demonstrated that the program could help such women improve their cognitive appraisals regarding the events they experience as they give birth in cross‐cultural settings, and that it can help them perform proper coping behaviors. Moreover, at 1 month postpartum, 36 of the 38 participants did not report experiencing stress from cross‐cultural stressors, demonstrating that our program may be able to help reduce cross‐cultural stress. Regarding postpartum depressive symptoms, the percentage of participants who presented with depressive symptoms 1 month postpartum was 21.1%, identical to the rate observed in the pre‐NIP period. Considering our findings, the NIP, which considers cross‐cultural difficulties, may be capable of reducing the stress caused by cross‐cultural stressors and preventing the onset of postpartum depression. This result could be built upon, as the NIP could be applied to foreign women with diverse cultural backgrounds other than Chinese. Moreover, as the number of Chinese immigrant women continues to increase, it may be necessary to identify a possible method of applying this program to clinical practice in Japan, where there are few Chinese medical practitioners.

## DISCLOSURE

No conflict of interest has been declared by the authors.

## AUTHOR'S CONTRIBUTIONS

QJ, EM, AS made substantial contributions to conception and design or acquisition of data or analysis and interpretation of data; QJ, EM, AS were involved in drafting the manuscript or revising it critically for important intellectual content; QJ, EM, AS gave final approval of the version to be published. Each author participated sufficiently in the work to take public responsibility for appropriate portions of the content; QJ, EM, AS agreed to be accountable for all aspects of the work in ensuring that questions related to the accuracy or integrity of any part of the work are appropriately investigated and resolved.
